# COVID-19 vaccination strategies depend on the underlying network of social interactions

**DOI:** 10.1038/s41598-021-03167-1

**Published:** 2021-12-15

**Authors:** Helena A. Saunders, Jean-Marc Schwartz

**Affiliations:** 1 Department of Analytical Chemistry, University of Vienna, Vienna, Austria; 2 School of Biological Sciences, University of Manchester, Manchester, UK

**Keywords:** Computational biology and bioinformatics, Systems biology

## Abstract

Since the onset of the coronavirus disease 2019 (COVID-19) pandemic, different mitigation and management strategies limiting economic and social activities have been implemented across many countries. Despite these strategies, the virus continues to spread and mutate. As a result, vaccinations are now administered to suppress the pandemic. Current COVID-19 epidemic models need to be expanded to account for the change in behaviour of new strains, such as an increased virulence and higher transmission rate. Furthermore, models need to account for an increasingly vaccinated population. We present a network model of COVID-19 transmission accounting for different immunity and vaccination scenarios. We conduct a parameter sensitivity analysis and find the average immunity length after an infection to be one of the most critical parameters that define the spread of the disease. Furthermore, we simulate different vaccination strategies and show that vaccinating highly connected individuals first is the quickest strategy for controlling the disease.

## Introduction

The coronavirus disease 2019 (COVID-19), caused by the severe acute respiratory syndrome coronavirus 2 (SARS-CoV-2), has spread globally since its identification in December 2019 and caused large amounts of life-threatening disease and deaths. Despite harsh limitations in economic activity and social contacts in many countries, its spread has generally not been contained and several countries or regions have been experiencing a succession of infection peaks or “waves”. An elimination strategy increasingly appears unrealistic at the global level and subsequent waves remain likely to occur in various locations for some time to come^1^. The expectation that infection spread could be stopped by herd immunity has been largely abandoned, given the high human cost involved and uncertainties on the persistence of immunity in recovered individuals, which is further compounded by the appearance of new viral variants^2–4^. Therefore, the focus is now turning towards vaccination as the best strategy to durably suppress the pandemic.

Computational models have been widely used since the onset of the pandemic to predict the spread of the disease, evaluate the effects of various suppression or mitigation scenarios, and assist governments to make decisions^5–7^. Two main types of models have been developed: the most common models are based on continuous and deterministic equations which assume homogenised and randomised spread through a population^8–10^. These models may be refined using different types of compartments that distinguish between age groups, activities, localisation, etc.^11^. The other type of models is agent-based and stochastic, where each individual is modelled by a node in a network of interactions. The properties of the network can be inspired from social sciences and take into account the known distribution of contacts in a population^12–16^. The exact network topology can be varied in different simulations, and even with a fixed network topology repeated simulations may still lead to varying results given the stochastic nature of the process, which provides a broader coverage of the range of observed outcomes in different locations. These models also better account for observed effects such as superspreading, which can be linked to the scale-free structure of social contact networks.

There is a high level of uncertainty on the level and duration of effective immunity to COVID-19 in populations and on how these parameters will affect the number of vaccinations required to suppress the disease. It is therefore timely to extend epidemic models to account for such effects and be able to assess different future scenarios^17^. Different approaches and experimental models can be used to measure immunity to SARS-CoV-2 and may reach different conclusions^18^. Antibody levels appear to decline drastically after 3 months, but the memory effect in T and B cells may last much longer and initiate a quicker immune response upon re-infection^19,20^. Studies of reinfections by other coronaviruses showed that individuals frequently become reinfected by the same seasonal virus at 12 months intervals and sometimes as early as 6 months^21^. There is growing evidence that reinfections can occur with SARS-CoV-2 too^22,23^, which may be further facilitated by the appearance of new variants of the virus^3^.

It is therefore timely to extend network-based epidemic models to account for different scenarios of waning immunity. Given the high level of uncertainty on immunity duration and effectiveness, different functions and parameter values should be investigated. At the same time, considering the underlying social network structure is critical to assess the effect of different vaccination strategies.

## Methods

### Network generation

We used a network science approach^24^ to represent interactions between individuals. We generated networks of 10,000 nodes (i.e. individuals) to represent an interacting community. Nodes are connected to other nodes via edges (i.e. interactions). Nodes that share the same edge are referred to as neighbors. The number of neighbors of a given node is referred to as the node degree. Not all nodes have the same number of neighbors. In fact, the node degree distribution in social networks has been shown to follow a power law^14,25,26^, whereby the majority of nodes have few connections and a few nodes have many connections. Networks that follow a power law distribution are known as scale-free networks. Nodes with many neighbors are referred to as hubs.

500 networks consisting of 10,000 nodes were generated using the *Static_Power_Law* method in the *igraph* package (Version 0.8.2) in Python (Version 3.8.5). Networks were generated such that the degree exponent of the node distribution fell between 2.0 and 3.2 and that their transitivity fell between 0.0 and 0.2, to represent realistic community interactions^25,26^. All networks were set to have less than 2,000,000 edges.

All nodes are initially in a susceptible state. We then randomly select a patient zero from the population to become infected. When a node catches COVID-19 it enters an infected state. A node that is in an infected state can pass the disease to any of its neighboring nodes. Once a node has recuperated from the disease it enters a recovered state, where it can no longer receive or pass on the disease. As the immunity of the disease wears off the node re-enters a susceptible state. An example network outlining the three different states is shown in Fig. S1. We do not consider deaths in our model as this would simply reduce the overall network size over time and is unlikely to affect the disease trajectory in the remaining network. We later introduce the concept of vaccination in our model. Assuming that the vaccine reduces the probability of a reinfection considerably, a vaccinated node immediately enters a recovered state and does not return to a susceptible state.

The probability with which the disease is passed from one node, the duration of the disease, and the probability of becoming reinfected after having had the disease are model parameters that we implemented as outlined in the following section.

### SIRS epidemiological model

**Figure 1:**
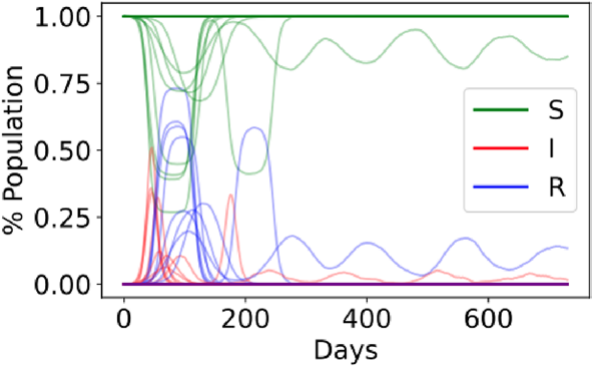
Example time courses of a susceptible-infected-recovered-susceptible (SIRS) epidemiological model ran on 10 randomly selected networks, showing various disease trajectories over 2 years. Individuals that are in the susceptible state at a given time are shown in green, those in a recovered state are shown in blue and those in an infected state are shown in red. The default model parameters as outlined in Table 1 were applied, with the exception of the immunity length (I) which was set to 63 days instead.

We ran a susceptible-infected-recovered-susceptible (SIRS) epidemiological model on the generated networks (Fig. S1). Our setup is similar to the SIR model previously described in^14^; however, here we did not consider individuals to recover permanently but to return to a susceptible state after the immunity of the infection wears off. A description of all default model parameters is outlined in Table 1. The default parameters were chosen to represent current knowledge on the spread of COVID-19 and are based on the following publications^19,21,27–30^. Wherever parameter estimation in the literature fluctuated vastly, as is the case with the length of immunity post infection^31^, we decided to air on the side of caution and selected a parameter value that would represent a realistic ‘worst case scenario’. We tested both a linear and logarithmic return to a susceptible state after an infection (Fig. S2) because both the length and the effectiveness of immunity are still an open discussion and have been shown to be highly variable parameters^19,28^. All our model results are based on 10 random simulations on each network using parameter values as described. A set of example time courses are shown in Fig. 1, showing the variety of possible disease trajectories. All our model results are based on time-courses that were run for 730 days (2 years).

**Table 1:** Default values of all parameters in the SIS epidemic model are outlined.

Parameter	Default value(s)	Name	Description
L	14	Length of infection	Number of days for which an individual that has contracted the disease can pass it on to others
p	[0.04, 0.4, 0.79, 0.71, 0.48, 0.32, 0.2, 0.08, 0.06, 0.04, 0.04, 0.04, 0.04, 0.04]	Probability of transmission	Probability of transmission over the two weeks of transmission after contracting the disease, such that p_i_ for i = [1:14]; i є Z. See Fig. S3 for a histogram
K	1	Transmission factor	Factor by which the probability of transmission is multiplied to vary the transmission intensity
I	90	Immunity length	Number of days that an individual remains immune from further reinfections after being infected
α_log_	16.8	Logarithmically dampened transmission	Probability of transmission is reduced by (K*δ) for n_i_ = n_1_, n_2_, … n_N_ days post infection and immunity and increases logarithmically such that δ_i_ = (α_log_ × ln(1 + n_i_)) / (1 + α_log_ × ln(1 + n_i_))
α_lin_	365	Linearly dampened transmission	Probability of infection is reduced by (K*δ) for n_i_ = n_1_, n_2_, … n_N_ days post infection and immunity and increases linearly such that δ_i_ = n_i_/α_lin_

The given initial values of the parameters shown in bold were varied by ± 30% for a sensitivity analysis.

### Parameter sensitivity analysis

We assessed the effect of both structural and epidemiological parameters on the outcome of the disease trajectory. To assess structural parameters (i.e. parameters that affect the underlying network) we analyse the degree exponents of the power-law distributions ranging from 2.0 to 2.3 and the transitivity of the networks ranging from 0.0 to 0.2 against the severity of the spread of the disease. We measure the severity of the spread of the disease as the maximum percentage of a population that is infected at one time, the percentage of the population that is infected 2 years after the initial onset of the disease, the maximum number of reinfection that occur to a single individual in the population and the percentage of days for which more than 1% of the population is infected over the 2 year period. We further varied the default epidemiological parameters (Table 1) by ± 30% and assessed the effect of each change in parameter against the severity of the spread of the disease.

### Vaccination strategies

Time courses were re-run as described in the above sections; in this section, however, selected nodes were vaccinated (i.e. remaining in a recovered state without returning to the susceptible state). We vaccinated either 5, 10, 20 or 30% of the population. In the first scenario all vaccinations were applied in a single day, one year after the first outbreak of the disease, and in another scenario vaccinations were applied starting one year after the first outbreak but evenly distributed over the time course of another year such that after two years either 5, 10, 20 or 30% was vaccinated. Two different vaccination strategies were considered in each of the previous scenarios. The first vaccination strategy randomly selects individuals from the population for vaccination, whereas the second strategy selects—in decreasing order—individuals with the highest amount of interactions in the networks for vaccination. We refer to the first strategy as “random vaccination” and the second strategy as “hub-first vaccination”.

## Results

### Both network structure and disease dynamics affect the disease trajectory

We analysed both network and model parameters, capturing the structure and the dynamics of disease transmission through a population, respectively. We assessed changes in these parameters against the following four outputs: (1) the maximum percentage of a population that is infected at one time, (2) the percentage of the population that is infected 2 years after the initial onset of the disease, (3) the maximum number of reinfection that occur to a single individual in the population and (4) the percentage of days for which more than 1% of the population is infected over the 2 year period.

A scale-free network is characterized by a power-law distribution f(x) = ax^−γ^^24,32^, where x is the node degree and f(x) the degree probability distribution. Thus, the critical parameter for defining the structure of such a network is the degree exponent, γ. While the degree distribution of the human interaction network is presumed to have a degree exponent between 2.2 and 3.2^25,26,33,34^, its exact value is debated^35,36^ and is furthermore condition-dependent on various lock-down and containment measures. An additional layer of complexity is added by the fact that degree distributions with the same degree exponent may have different community structures within and these too may change under various government regulations. These differences in clustering can be measured by the transitivity of a network, generally defined as C = tr(A3)/∑_i≠j_ (A2)_i,j_^32^.

**Figure 2:**
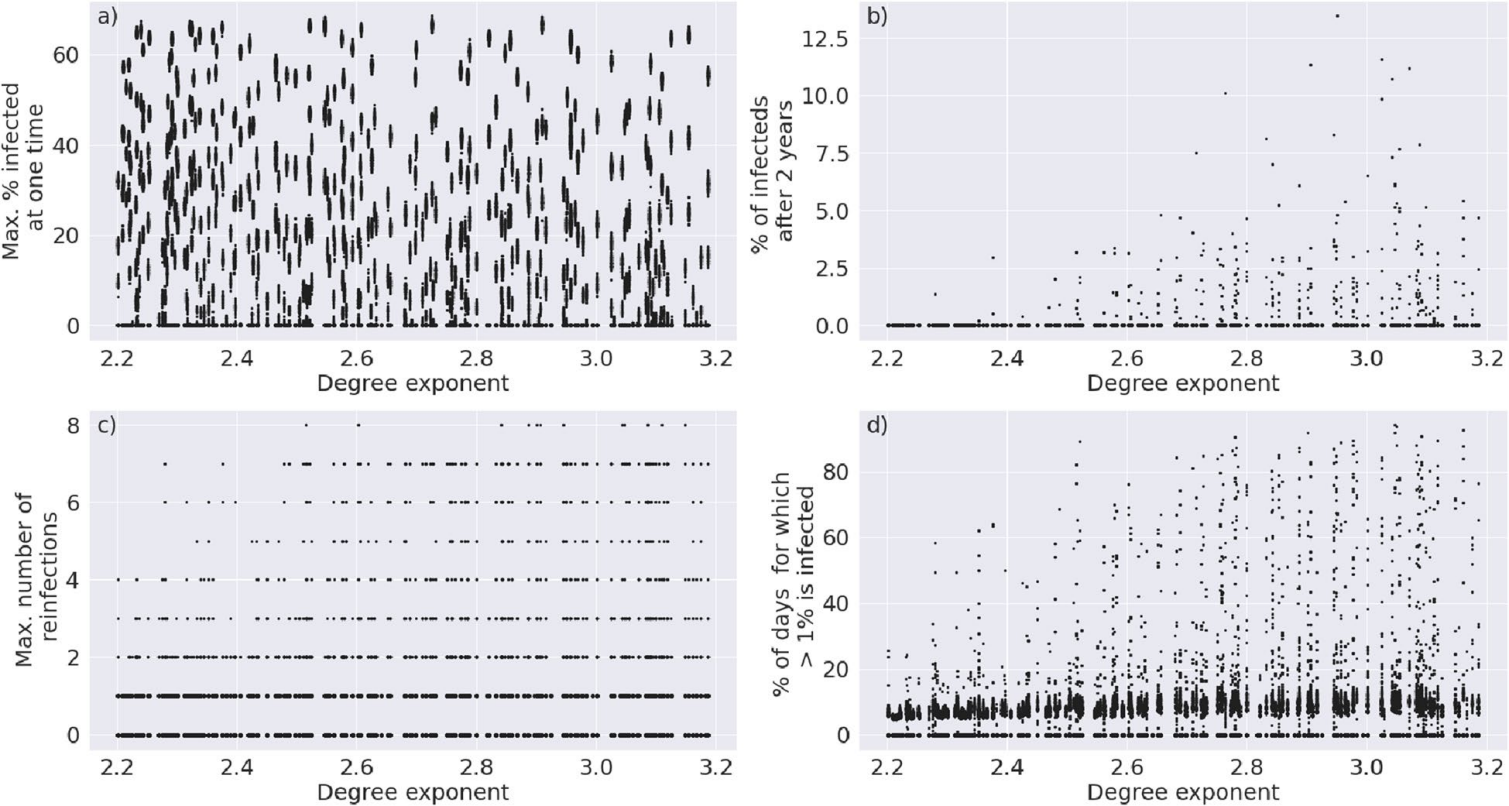
The degree exponent, γ, which determines the underlying structure of the interaction network, is plotted against (**a**) the maximum percentage of a population that is infected at one time, (**b**) the percentage of the population that is infected 2 years after the initial onset of the disease, (**c**) the maximum number of reinfection that occur to a single individual in the population and (**d**) the percentage of days for which more than 1% of the population is infected.

**Figure 3:**
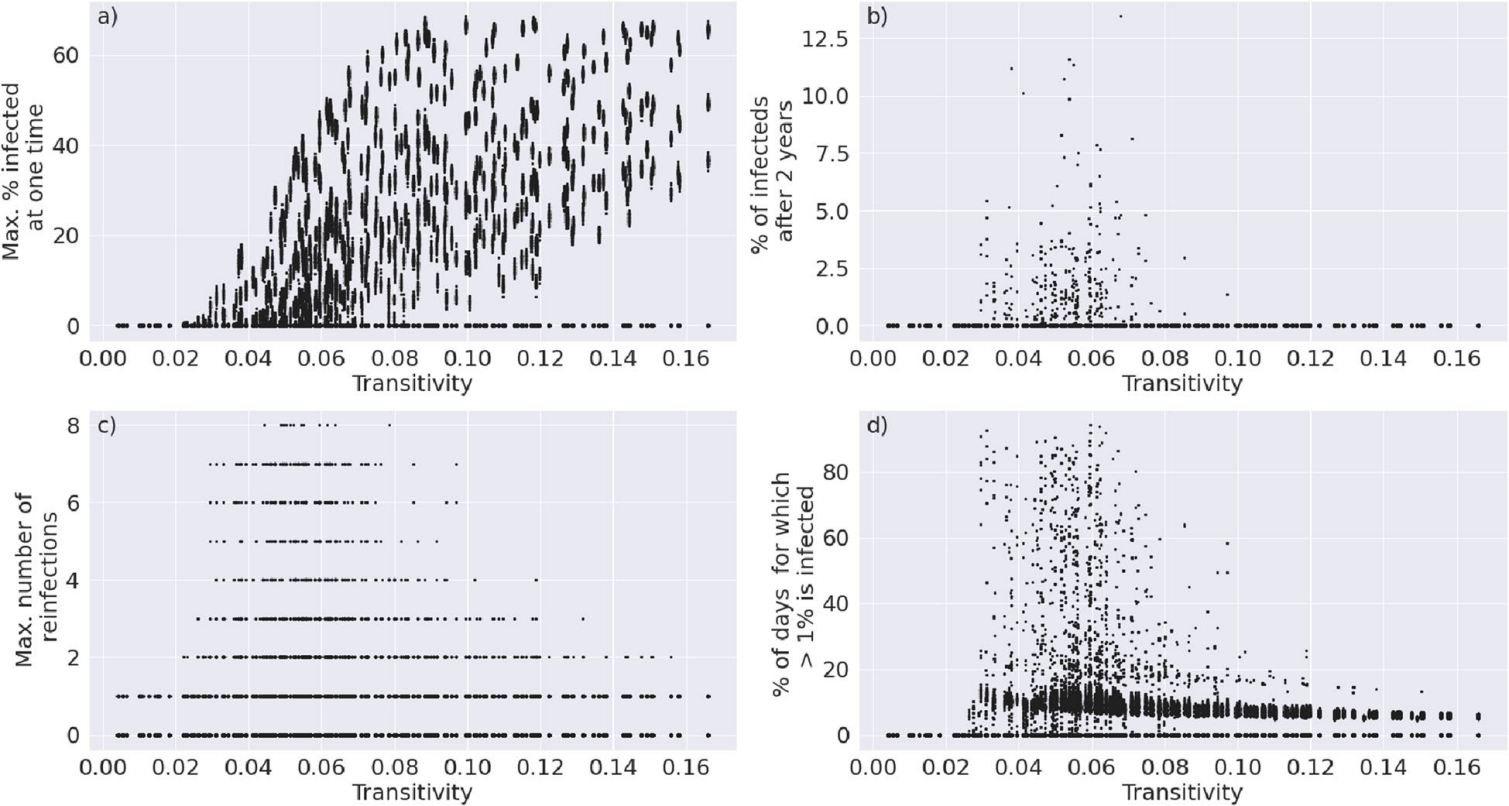
The transitivity, which determines the community structure of the interaction network, is plotted against (**a**) the maximum percentage of a population that is infected at one time, (**b**) the percentage of the population that is infected 2 years after the initial onset of the disease, (**c**) the maximum number of reinfection that occur to a single individual in the population and (**d**) the percentage of days for which more than 1% of the population is infected.

Upon generating 500 networks of 10,000 nodes, with a degree exponent between 2.2 and 3.2 and transitivity between 0.05 and 0.2, and running our SIS model on each of them, we were able to capture the effect of the degree exponent (Fig. 2) and of transitivity (Fig. 3) on the trajectory of the disease. A higher degree exponent means a lower variance in node degrees. Figure 2b,d show that when the variance in node degrees is extremely high (i.e. the degree exponent is low), then the disease is more likely to die out early; in this scenario, the disease never infects a hub^14^. If, however, the transitivity of the network is very high, the likelihood of a pandemic is also reduced (Fig. 3); this is because in a highly clustered network the disease is more likely to die out in a local cluster before spreading to the wider network. As a result, disease propagation through networks with an intermediate degree exponent and an intermediate transitivity tends to experience a severe spread of the disease, whereby the majority of individuals get infected.

The transitivity of a network is correlated to the number of edges used to generate the network. Comparing Fig. 3 to Fig. S4, the latter highlighting the effect of the number of edges on disease trajectory, shows that edge number alone cannot account for the observed patterns in disease trajectory. For example, the maximum number of infected individuals at a time increases linearly with the number of interactions in the network (Fig. S4a). While a higher transitivity does increase the maximum number of infected individuals at a time, this effect levels off at a transitivity of around 0.075 when the disease outcome is most variable (Fig. 3a).

**Figure 4:**
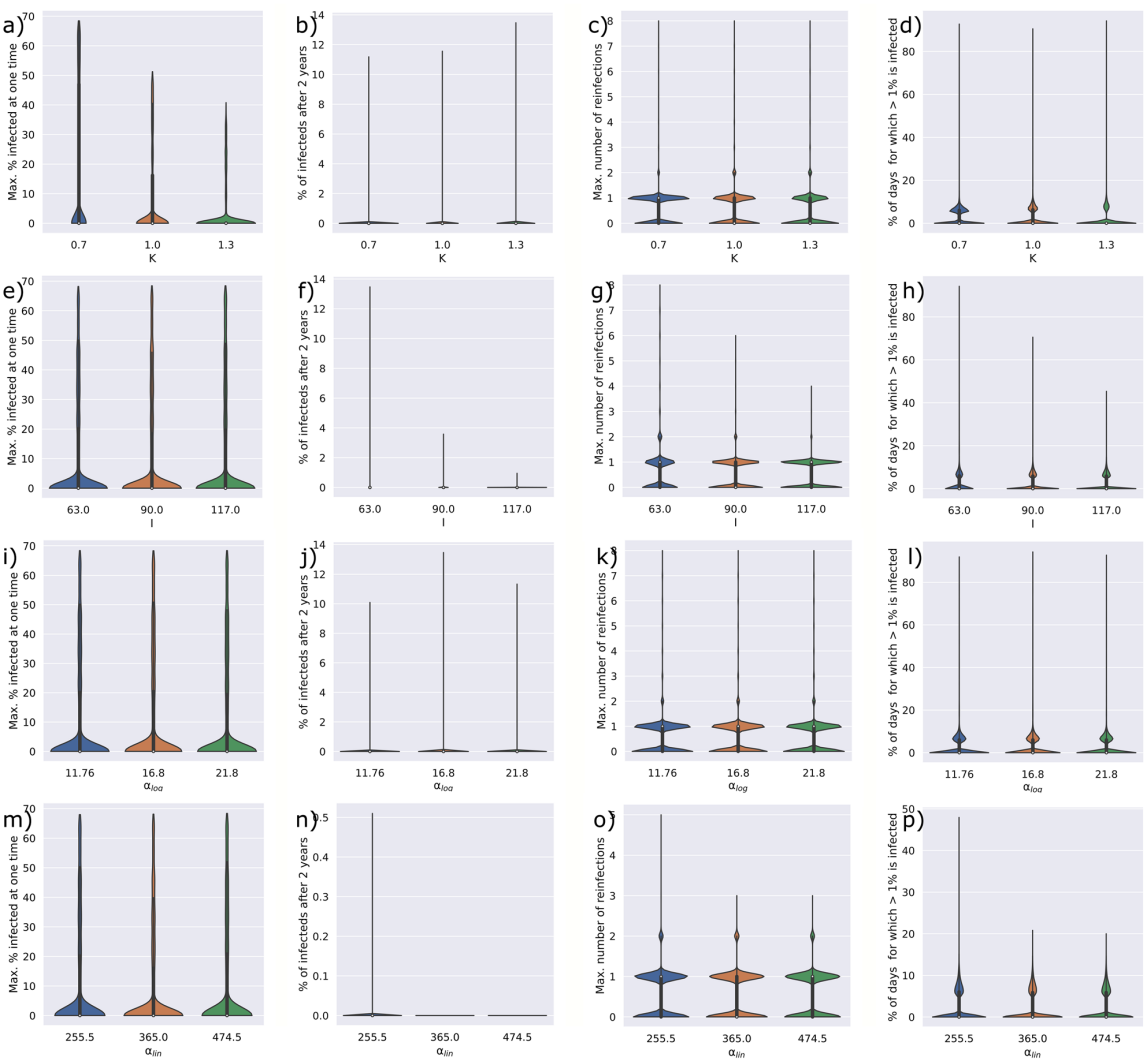
Parameter sensitivity analysis of the transmission factor—K (**a**–**d**), the immunity length—I (**e**–**h**), the logarithmic dampening—α_log_ (**i**–**l**), and the linear dampening—α_lin_ (**m**–**p**). Default parameters were varied by ± 30% and the effect on the maximum percentage of the population infected at a time (a,e,i,m), the percentage of infected individuals after 2 years (**b**,**f**,**j**,**n**), the maximum number of reinfections (**c**,**g**,**k**,**o**), and the percentage of days for which more than 1% of the population is infected (**d**,**h**,**l**,**p**) is shown.

In addition to the structural parameters, we analysed parameters that define the disease dynamics (i.e. the rate of transmission). A detailed description of all model parameters is provided in Table 1 of the Methods. Here, we analyzed the influence of the probability of transmission, adjusted by the transmission factor K, the length of immunity after infection I, and the return to becoming a fully disease transmittable individual after infection and immunity. Two scenarios for infected individuals to become again susceptible were considered: logarithmic dampening, whereby susceptibility increased rapidly immediately after infection, characterized by α_log_ (Fig. S3) and linear dampening, whereby susceptibility increase linearly upon infection, characterized by α_lin_ (Fig. S3). Figure 4 shows the critical points of disease trajectory when these model parameters are altered by ± 30%. We observe that changes in the I and the α_lin_ parameter have the greatest effect on the disease outcome, affecting the number of days that more than 1% of the population is infected, the maximum number of reinfection in the population, and the number of infected individuals after 2 years. The combination of these parameters determines how long, after an infection, an individual is again able to transmit the disease. Given that the logarithmic dampening returns individuals to the susceptible state quicker (Fig. S2), the α_lin_ parameter has a greater influence over the disease outcome than the α_log_ parameter (Fig. 4).

Interestingly, the probability of transmission affects the number of individuals that are infected at one time (Fig. 4a) but has little effect on the other disease outcomes such as the duration of the disease and the possible number of reinfections by any individual. In fact, the probability of transmission is the only parameter that has an effect on the number of individuals infected at one time (Fig. 4a,e,i,m).

### Vaccination strategies that target hubs are most effective

**Figure 5:**
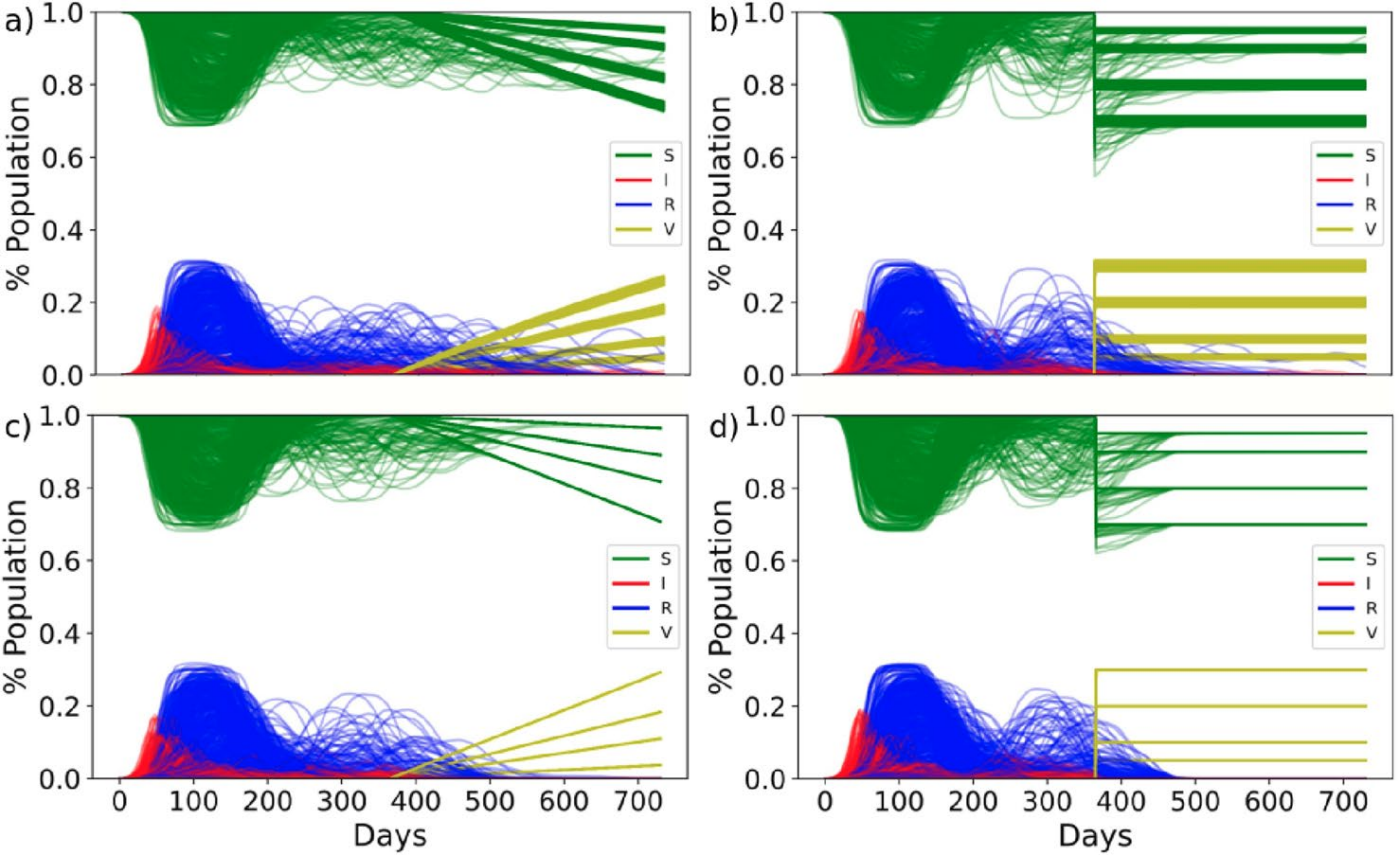
Time courses of percentage of infected (red), recovered (blue), susceptible (green) and vaccinated (yellow) individuals in the population. Vaccines were administered 1 year after the outbreak of the disease to either 5, 10, 20 or 30% of the population. Vaccines were administered in a single day (**b**,**d**) or over evenly distributed over 1 year (**a**,**c**). Vaccines were administered randomly (**a**,**b**) or priority was given to hub nodes with the highest number of interactions in the network (**c**,**d**). Model parameters were set to the default parameters outlined in Table 1.

Keeping the default model parameters (Table 1) we again ran time courses of the disease transmission through the network for 2 year (730 days). This time, however, we “vaccinated” a percentage of the population—either 5, 10, 20 or 30%—after 1 year (Fig. 5). Assuming the vaccine to be 100% effective, vaccinating an individual is the same as removing the individual from the network. All four percentage vaccinations are shown on the same graph to give an overview of the range of outcomes; evidently vaccinating 30% of the population has a greater potential to limit the spread of the disease than 5%. We then consider two modes of vaccine applications: one where the selected percentage of individuals is vaccinated all on one day and one where the selected percentage of individuals is vaccinated over the course of one year. We further consider two vaccination strategies: one where the individuals are chosen randomly for vaccination and one where individuals with the most interactions in the network (i.e. hubs) are vaccinated first.

Figure 5 shows the hub first vaccination strategy (Fig. 5c,d) to be more effective than the random vaccination strategy (Fig. 5a,b) in that the disease dies out within the first 300 days of when the first vaccine is administered. Evidently, administering all vaccinations in one day (Fig. 5b,d) is more effective than administering event space over one year (Fig. 5a,c); evidently, this application is unrealistic to implement and should be considered as a most favourable scenario, but it nevertheless remains less effective than the hub-first vaccination strategy (Fig. 5b,c).

**Figure 6:**
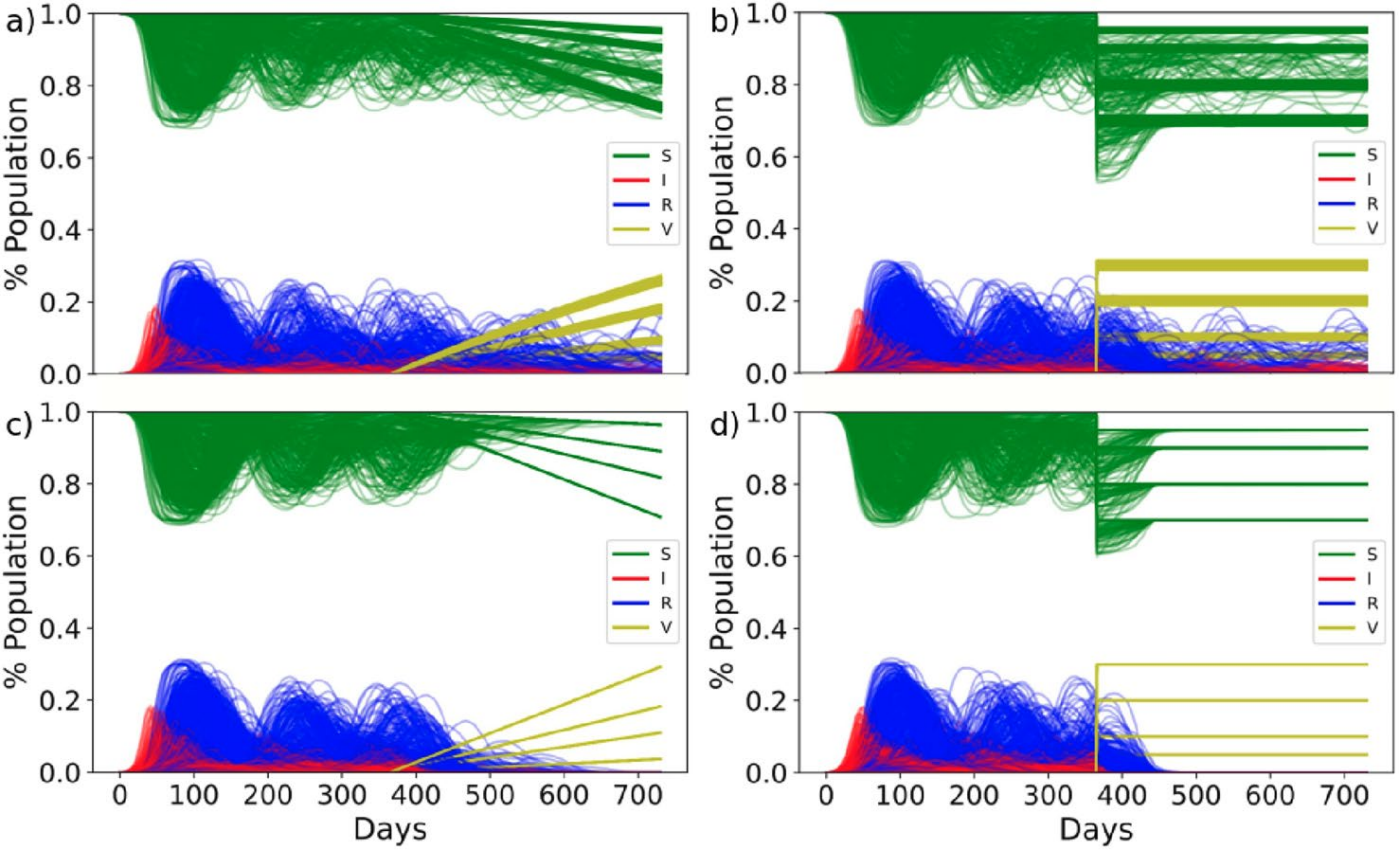
Time courses of percentage of infected (red), recovered (blue), susceptible (green) and vaccinated (yellow) individuals in the population. Vaccines were administered 1 year after the outbreak of the disease to either 5, 10, 20 or 30% of the population. Vaccines were administered in a single day (**b**,**d**) or over evenly distributed over 1 year (**a**,**c**). Vaccines were administered randomly (**a**,**b**) or priority was given to hub nodes with the highest number of interactions in the network (**c**,**d**). Model parameters were set to the default parameters outlined in Table 1, with the exception of the immunity length which was set to 63 days. Only networks with a transitivity between 0.5 and 0.8 were considered.

**Figure 7:**
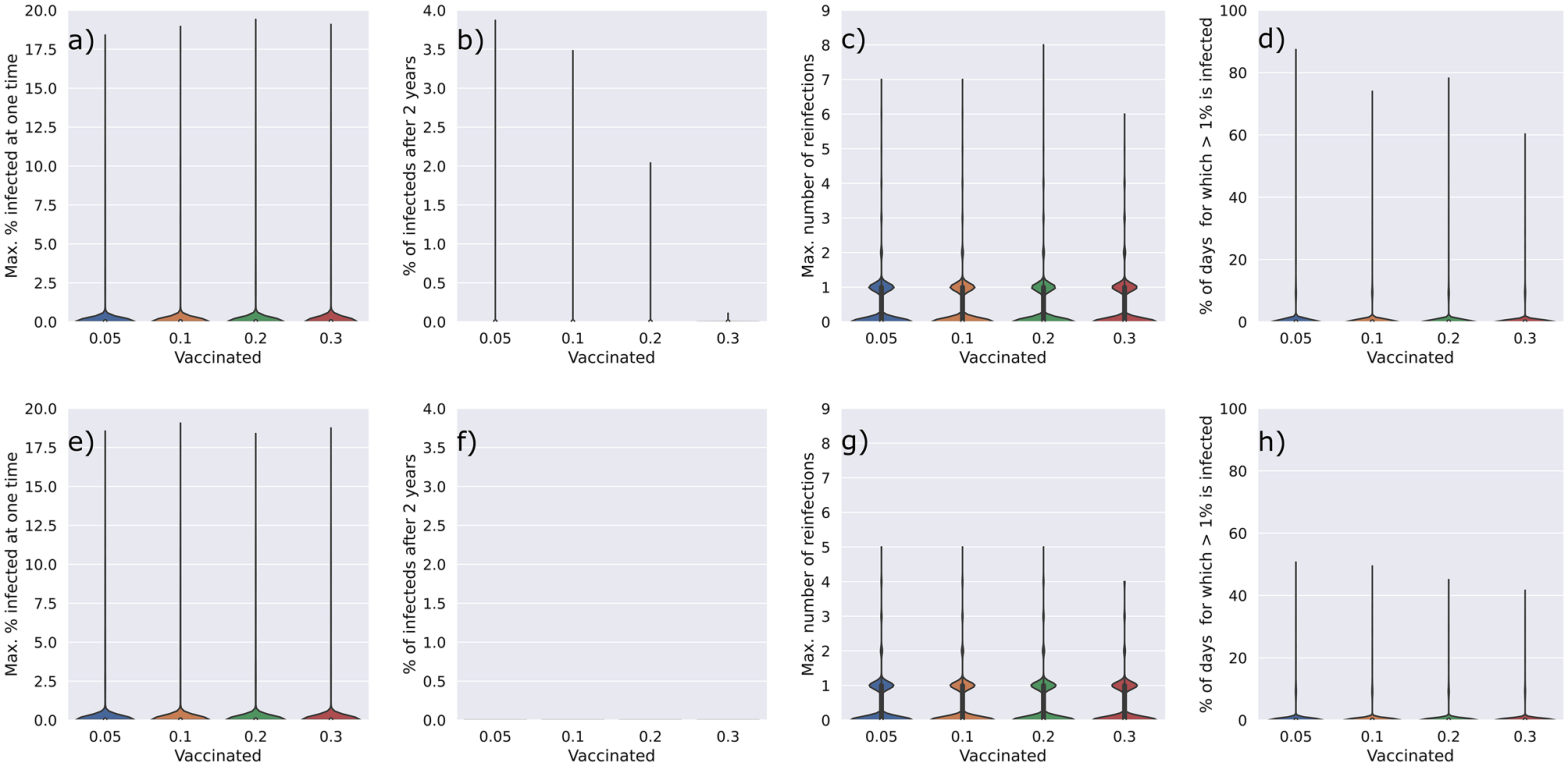
Summary data of the time courses shown in Figs. 5 and 6 comparing random (**a**–**d**) versus hubs first (**e**–**h**) vaccination strategies. The maximum percentage of the population infected at a time (**a**,**e**), the percentage of infected individuals after 2 years (**b**,**f**), the maximum number of reinfections (**c**,**g**), and the percentage of days for which more than 1% of the population is infected (**d**,**h**) is shown. In (**f**) all data points are zero.

Since our previous results on the model parameters highlighted the immunity length after an infection to be one of the most sensitive epidemiological parameters and transitivity to be a sensitive structural parameter, we re-ran the vaccination results but with the immunity length set to 63 instead of 90 days, and selected only networks with a transitivity between 0.5 and 0.8 (Fig. 6a–d). As expected, reduced immunity and intermediate transitivity results in a worse spread of the disease, such that random vaccination cannot eliminate the disease (Fig. 6a,b). We summarized Figs. 5 and 6 using our previously established measurements for disease severity (Fig. 7a–h) and show that only in the vaccination strategies that consider hubs first can the disease be entirely eliminated (Fig. 7b,f). The hub first vaccination strategy also clearly reduced the total number of days for which more than 1% of the population is infected at one time (Fig. 7d,h).

## Discussion

We, and others, have previously shown that considering the underlying network of human interactions is crucial to identifying the most effective strategies for managing and containing a pandemic^11,13,14,36^. Many of the models currently used to predict the COVID-19 pandemic do not take into consideration the structure of the underlying human interaction network. Models based on differential equations or random diffusion^8,10,37^ typically assume that human interaction behaviors are homogenous. Failure to consider the underlying human interaction network, which is not homogenous and not random, limits the range of possible mitigation strategies that can be considered^15^.

While there is data available that can be used to characterize the underlying network structure of human interactions, these data sets are largely regionalized and often do not account for long-range interactions and connectors between different clusters^38^. Using three different types of COVID-19 models—exponential growth, self-exciting branching, and an SIR model, Bertozzi et al*.*^39^ showed that model parameters can vary significantly for a given location and can carry a high uncertainty. It is therefore pertinent to consider a level of uncertainty, not only in the parameters that define the spread of the disease, but also in the parameters that define the underlying interaction network to account for a range of societies^40^.

Our results show that a high transitivity increases the number of infected individuals at a time. This is because the disease is very quick to spread within well-connected local clusters. However, in networks with a high transitivity the disease is also less likely to spread from one cluster to another, as most of the connections lie within and not between clusters. While many government mitigation strategies have emphasized a “local social bubble” approach, the approach is most effective only when the network transitivity is increased as a result and the total number of connections between and within local communities is actively limited. This strategy closely aligns with the link removal analysis conducted by Bellingeri et al.^41^ and proposed as an effective non-pharmaceutical intervention for controlling the spread of COVID-19. Characterizing nine possible stages of infection, Giordano et al.^42^ used a deterministic model to show that non-pharmaceutical interventions hold a higher potential for epidemic control than vaccination strategies alone.

In addition to the structural parameters of the network, we further analysed the sensitivity of the model parameters that are defined by the virus itself (i.e. its infectiousness, its virulence and the immunity that can be achieved against it). As new mutations and new data around the infectiousness and immunity of the COVID-19 virus emerge^2,4^, model parameters will have to be measured, re-calculated and tuned accordingly. Our sensitivity analysis of the model parameters shows that the level of infectiousness (i.e. the probability of the disease being transmitted) greatly affects the height of the initial peak of infected individuals. When it comes to the long-term spread of the disease after the initial peak, the obtained immunity is, however, a far more relevant parameter. If individuals can maintain an average immunity of three months rather than two months, the disease is far more likely to be eradicated. Given that the length of immunity after an infection is still highly debated^19–21,43^ and has yet to be confirmed for new strains, it will be critical to confirm this parameter as soon as possible in order to improve the predictive power of COVID-19 models.

Finally, we implemented different vaccination strategies into our model and, in line with previous research^14,36,44,45^, show that targeted vaccination of hubs is far more effective than random vaccination. Multiple susceptible-infected-recovered-vaccinated (SIRV) models or variants thereof have emerged in the recent literature^46–49^; however, most of them are deterministic. Using a stochastic model that considers the underlying social interaction network, we show that hub-specific vaccination strategies are able to half the number of reinfections and the number of days for which more than 1% of the population is infected. Slight errors in model parameters, such as an overestimation of the length of immunity for infected individuals, could drastically affect the estimated ability of a vaccination strategy to eradicate the disease. Selecting vaccination strategies that are more robust to changes in the biological and structural parameters of the model is therefore far more likely to be successful. In fact, Giubilini et al.^50^ argue that there are strong ethical reasons to vaccinate the young (who are more likely to be hubs) to achieve herd immunity quicker and to protect the vulnerable. Evidently, there are other operational and ethical challenges that may need be considered to effectively implement a hub vaccination strategy, including the identification, prioritization and willingness of highly connected individuals^51,52^. Vidondo et al.^53^ have already developed a strategy for identifying potential super-spreaders.

Other vaccination strategies which we did not consider in our model, is the “vulnerable first” vaccination strategy^54^, currently employed by many countries. As we did not consider vulnerable versus non-vulnerable nodes in our network, this vaccination strategy defaults to be the same as a random vaccination strategy. Another possible vaccination strategy is the so-called “ring vaccination” strategy, whereby neighbors of a node that has been identified to have the disease are vaccinated first^55^. While this is an interesting concept, Tetteh et al.^55^ assume that individuals who have had the disease cannot get re-infected and are therefore likely over-estimating the vaccination requirements for herd immunity. While our model results account for possible re-infections, we do assume that vaccinated individuals maintain lasting immunity. Recent literature has shown a steady decline of antibody level in vaccinated individuals^56,57^. Nevertheless, our hypothesis remains reasonable as lasting immunity can be achieved through repeated administration of vaccines. Building on our model framework, it may be worth including an immunity depreciation function in future works. This would account for a scenario where booster vaccines cannot be administered fast enough*.*

Elderly people, especially those with pre-existing conditions and comorbidities have been shown to be particularly susceptible to COVID-19, affecting both disease progression and outcome^58–60^. The stochasticity of our model (i.e., the probabilistic event of getting infected) inherently accounts for heterogeneous populations. If, however, the population at hand primarily consists of elderly or vulnerable people, the parameters with which we have tuned our model may need to be adjusted to accurately reflect the population average.

In conclusion, our research shows how different network properties and different virus properties affect the spread of a disease in a social interaction network. To predict accurate outcomes of vaccination strategies, it is crucial to assess how the network structure is changing as a result of either enforced or voluntary behavioural changes in the population, and to fine-tune model parameters to mutant-specific biological parameters, such as the probability of infection and the length of the achieved immunity after an infection. Given our analyses, a hub-first vaccination strategy is clearly more effective than a random vaccination strategy, highlighting why it is pertinent to consider the underlying social structure as well as heterogeneity within a given population, as we have done here. While this result may have direct implications on vaccination policies, we have further shown that the probability of infection is one of the most sensitive parameters when considering network models of COVID-19. It is therefore recommended that to accurately parametrize strain- and population-specific models and to strengthen the validity of future model predictions, a special research emphasis is placed on determining the probability of infection and how this is affected by the emergence of new viral mutations.

## Supplementary Information

Supplementary Information.

## Data Availability

The code for all of the analyses included in this manuscript is publicly available on GitHub at https://github.com/HAHerrmann/NetworkEpidemics and under the following Zenodo https://doi.org/10.5281/zenodo.4485325.
