# Supplementary material for: COVID-19 vaccination strategies depend on the underlying network of social interactions
Source: Sci Rep. 2021 Dec 15;11:24051. doi: 10.1038/s41598-021-03167-1 (PMC8674282; doi:10.1038/s41598-021-03167-1)
Supplement: Supplementary file 1 — Supplementary Information. [file 41598_2021_3167_MOESM1_ESM.docx]

**Supplementary Information**


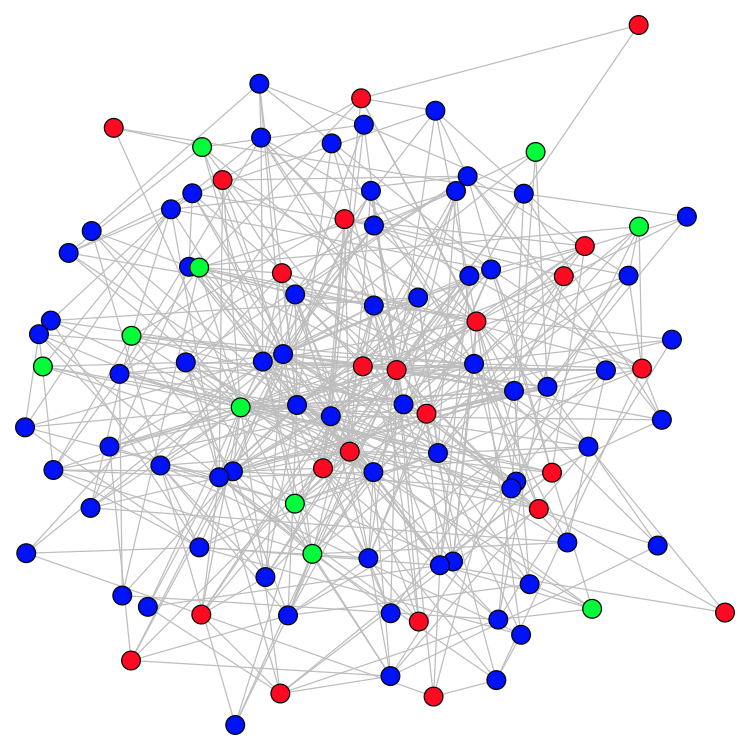


**Figure S1:** Illustrative example of a network and its possible states: infected (red), susceptible (green) and recovered (blue). This toy network has 100 nodes and 400 edges, a degree exponent of 3.2 and a transitivity of 0.18.


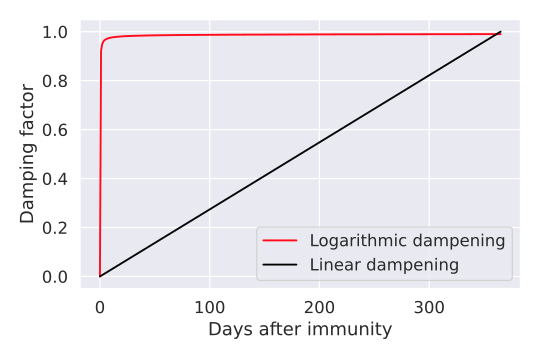


**Figure S2**: Dampening factor δ by which the probability in infection is multiplied for individuals that have previously contracted the disease.


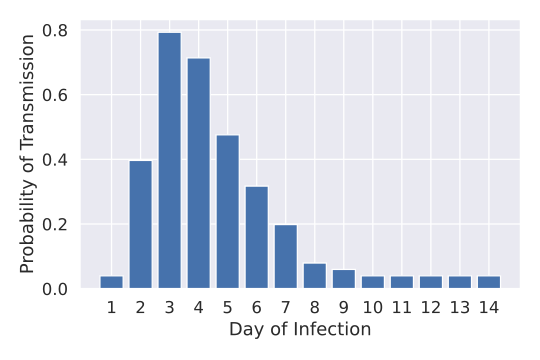


**Figure S3**: Histogram of the default probability of transmission of the disease over 14 days.


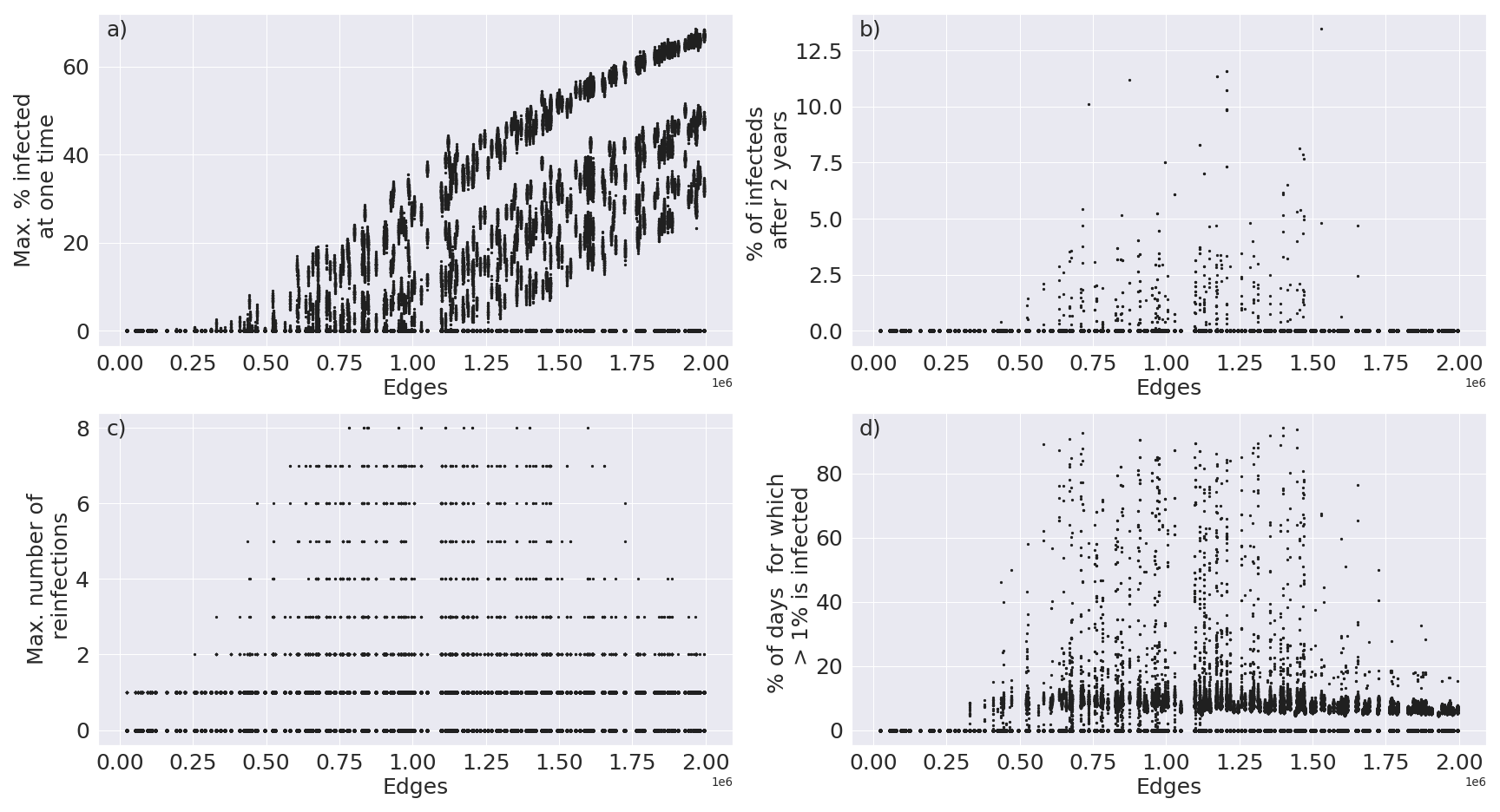


**Figure S4:** The number of edges (interactions) in a network are plotted against (a) the maximum percentage of a population that is infected at one time, (b) the percentage of the population that is infected 2 years after the initial onset of the disease, (c) the maximum number of reinfection that occur to a single individual in the population and (d) the percentage of days for which more than 1 % of the population is infected.
